# An assessment of the influence of personal branding on financing entrepreneurial ventures

**DOI:** 10.1016/j.heliyon.2019.e01164

**Published:** 2019-02-06

**Authors:** Suzanna ElMassah, Ian Michael, Reynold James, Ionica Ghimpu

**Affiliations:** a College of Business – Zayed University, United Arab Emirates; b Faculty of Economics & Political Science – Cairo University, Egypt; c Emirates Airlines, United Arab Emirates

**Keywords:** Business, Economics, Industry, Information science, Political science, Sociology

## Abstract

This research explores the influence of an entrepreneur's personal brand in attracting capital, by examining the validity of the Entrepreneurial Brand Personality Equity (EBPE) model of Balakrishnan and Michael (2011). Its particular concern is whether investors provide funding to an entrepreneur's idea, or, to the entrepreneur behind the idea. Concomitantly, it seeks to identify the variations in the importance accorded by different investors to the several variables of the EBPE model, and whether these variations-and also the stages of business-influence the final investment decisions of investors. The findings of this mixed methods study hold significant implications for various stakeholders, and suggest that the presence of the EPBE model's dimensions in an entrepreneur are very necessary for attracting investors' capital. The personal branding of the A-team in particular, clearly emerged as the most critical variable of the EBPE model, based on the type of investor and stage of the entrepreneurial venture.

## 1 Introduction

According to the latest Global Entrepreneurship Monitor report (GEM, 2016-17), the current number of entrepreneurs across the globe is 582 million (see Kelley, 2017), which is a significant increase (of 45.5%) over the 400 million that existed in 2011 (Bosma et al., 2011). Interestingly, the majority of these are early-stage entrepreneurs. This corroborates the assertions of the extant literature on entrepreneurship, regarding new business creation being the most critical driver of economic growth, social development and the competitiveness of nations, and about the vital role entrepreneurs play in the global economy by developing new businesses, creating jobs, spurring economic activity and driving innovation (Domingo, 2017; GEM, 2016-2017; Ernst and Young, 2011; Wennekers et al., 2005; Van Stel et al., 2005; Audretsch and Keilbach, 2004; Wennekers and Thurik, 1999). Therefore, the special interest that has been evinced in the processes leading to the establishment of new enterprises across the world (Khoury and Prasad, 2015; Wright and Stigliani, 2012).

Despite the foregoing acknowledgement of the role played by new and small businesses globally, what ironically persists, is the ‘perennial problem’ (p29) of access to finance for small businesses (GEM, 2016-17), with those worst hit by this limitation being the early stage ventures (Frydrych et al., 2014). This, as they are often bereft of credit histories -or assets to serve as collateral- required to secure loans from financial institutions, to fund their potential entrepreneurial ventures (also see Kew et al., 2013). Whilst this is a critical issue, since the availability of funding significantly influences both, the level and the type of entrepreneurship prevalent in a given economy, its ‘perennial’ nature is borne out by Stinchcombe (1965), who lamented thus over half a century ago:

‘Entrepreneurs are wealth constrained and cannot raise debt to pay for resources needed, as the venture suffers from liability of newness’

The issues associated with early stage and young entrepreneurs' access to capital is well documented in the literature. A key reason for this is that such firms are typically not yet profitable and lack tangible assets, which nullifies debt financing as an option for them (Denis, 2004). They also lack the prerequisites for listing in the financial markets, which precludes their ability to rely on issuance of securities to finance their ventures. Resultantly, according to Bhide (2000) and Chemmanur and Fulghieri (2014), their sources of funding are restricted to Venture Capitalists (VC's), Angel Investors (AI's) and Private Equity Investors (PEI's). Here too, however, owing to the Global Financial Crisis of 2008 and the consequent increase in bankruptcy rates amongst entrepreneurs, there has been a diminishing of VC's and AI's appetite for new venture financing (Mazzarol, 2012), given the inherent risks involved. This has further exacerbated the situation.

The foregoing has eventuated in a highly contested playfield, with several aspiring entrepreneurs jostling to win favor of the few investors available to finance their ideas. Intrinsic to this situation is the objective of our paper, that examines whether investors provide funding to the entrepreneur's idea, or to the entrepreneur behind the idea.

There are protagonists for either side of the aforesaid proposition. For example, while Mason and Stark (2004) claim that investors invest first in the idea and then in the entrepreneur, there are others who argue that entrepreneurs are represented by their ideas, and it is therefore the people behind ideas who execute the same, and convert the idea into a successful business (e.g., Macmillan, Siegel & Narasimha, 1985; Rose, 2014).

We extend this (latter) line of thinking and posit that if ideas are a reflection of the entrepreneur, the entrepreneur's personal branding would arguably reflect the future company brand (Montoya and Vandehey, 2003; Razeghi et al., 2016), which tantamounts to the entrepreneur's personal brand becoming a determinant for investors' decision making. Hence, the need for entrepreneurs to pay particular attention to the notion of ‘personal branding’ -first discussed in Tom Peter's (1997) article, in the management magazine Fast Company- so as to improve their competitiveness in successfully attracting capital from investors. This study addresses this aspect too.

Whilst the importance therefore of an entrepreneur's personal brand cannot be overstated (see Lair et al., 2005), there exists a paucity of research literature on the brand building strategies available to Small and Medium Enterprises (SMEs) and prospective entrepreneurs, particularly in the context of the nexus between personal branding and the attraction of funding.

Further, whereas there exists an abundance of literature on successful entrepreneurs, their characteristics, behavior, motivations, their strategies for success -and also reasons for failure- these elements have mostly been dealt with from the standpoint of venture success, business performance and economic gain (Makhbul and Hasun, 2011; Mitchell et al., 2002). Whilst these areas are indeed worthy of study, there is a void in the research concerning entrepreneurs' personal brands and brand building strategies (Ahonen, 2008), that are intertwined aspects (Shepherd, 2005). Further, although the literature on entrepreneurial personalities and traits that drive venture success abounds (see Mitchell et al., 2002), the same is patchy in terms of personality factors that investors consider when deciding whom to finance, barring a few studies (e.g., Macmillan et al., 1985). Here too, the level of importance of these factors is not clearly articulated, which is a deficiency that needs addressing.

A notable exception that has sought to address the foregoing gaps is the Entrepreneurial Brand Personality Equity (EBPE) model of Balakrishnan and Michael (2011), which specifically demonstrates the importance of various branding dimensions, to investors' decision making processes. This model was generated from research conducted during a major entrepreneurship event at Dubai (Celebration of Entrepreneurship, 2010), that attracted over 1500 attendees and 200 speakers from across the globe, comprising a mix of established and nascent entrepreneurs, investors, government facilitators, and incubators. Several VC's, incubators and people brand specialists were interviewed at the event.

The EBPE model was created based on the findings from these interviews, which confirmed the need for entrepreneurs to focus on certain factors that constituted their ‘entrepreneurial brand personality’, that investors considered when deciding to invest. EBPE is defined as the net worth of the projected capability of the entrepreneur in the marketplace (Balakrishnan and Michael, 2011). The model comprises three key dimensions: Brand Personality (BP), Halo Brands (HB), and Brand Value (BV), with each dimension associated with a set of variables (traits).

Since its conceptualization however, the applicability of the EBPE model has not been validated either through further quantitative or qualitative research studies. Further, no evidence exists of previous studies that considered all the EBPE model's dimensions, nor of any statistical analysis on the same, as components of an entrepreneur's personal brand. Resultantly, the model's efficacy from an investors' perspective- in relation to actual investments undertaken based on entrepreneurs' personalities- has not yet been ascertained. Arguably therefore, a significant possible benefit that would accrue to entrepreneurs by the validation of the EBPE model, would be their ability to make informed choices about brand building strategies most suited to attract investors' funding in the first instance, and to subsequently ensure the sustenance of such financing arrangements on an ongoing basis.

This study's objective is to examine the importance of the EBPE model^2^ (and its dimensions and variables) to investors' decision making as regards their amenability to financing entrepreneurial ventures. This is achieved by seeking answers to the following four research questions. (i) do the EBPE model's variables matter during investment related decision making (ii) do all types of investors value the model's variables similarly (iii) are the model's variables valued similarly at all the stages of business (iv) what are the implications of the EBPE model's variables for ensuring sustainable, ongoing finance for the entrepreneurial venture.

By answering these questions, this study unravels how investors assign importance to the different variables of the EBPE model. Further, it determines whether different types of private investors value these variables differently, and whether these variables are equally important at all the stages of investment. In so doing, this research attempts to provide quantifiable data demonstrating the level of importance of each of the model's variables to an investment decision, and how these levels vary with different investors, and also with the different stages of business. The study's results hold significant implications for both entrepreneurs as well as investors. Further, they serve to reduce the increasing rigor-relevance gap in entrepreneurship research highlighted by scholars such as Frank and Landstrom (2016), who opine that interesting studies must develop applicative knowledge and be relevant to practice, which arguably is the case with this study.

## 2 Background

The 18^th^ report of the G.E.M. (2016-17) reveals a surge in the number of entrepreneurships globally, and reiterates the established fact that accessing capital continues to remain a universal concern, particularly affecting young, early stage entrepreneurs. Strongly corroborating this, is an O.E.C.D (2012) report, about finance representing one of the most significant challenges for entrepreneurs, and for the creation, survival and growth of small businesses. This issue attains serious proportions -as resources are the heart of a firm's existence and growth (Reddi and George, 2012)- and has therefore garnered much attention within the entrepreneurship literature (George, 2005; Penrose, 1959), as with the case of the financial economics literature (e.g., Eckhardt et al., 2006; Gompers, 1995; Kaplan and Stromberg, 2003; Lerner, 1995). A logical question in entrepreneurship therefore, is about how entrepreneurs mobilize resources in the pursuit of an opportunity.

Whilst Reddi and George (2012) opine that entrepreneurs who are not wealth constrained may garner resources by paying for them with cash, the accepted assumption however, is that the entrepreneur is indeed wealth constrained, and requires capital from other sources, that are either debt financing or equity financing.

Aside from friends and relatives, entrepreneurs stand limited chances of receiving debt financing from traditional lending institutions such as banks. This owes to their absence of a demonstrated track record of business, financial viability and success (Berger and Udell, 1998), and concomitantly, the absence of information sought by traditional lenders to estimate the level of risk in financing entrepreneurs, and also their competence and commitment that have a bearing on the prospects of their proposed ventures (Binks and Ennew, 1996, 1997). Equity financing through Initial Public Offerings (IPOs) is largely infeasible too, in the absence of an entrepreneur's record of profitable operations, and/or of being in business for several years.

The foregoing impediments frequently constrain entrepreneurs to rely on three primary sources of outside equity financing: VC's, AIs, and PEI's respectively. There is a rich literature describing the specific roles of these players in the entrepreneur-financing process (Denis, 2004), and also the differences between their approaches to making decisions on the same (Kerr et al., 2014; Goldfarb et al., 2007; Shane, 2008; Mitteness et al., 2012; Lamoreaux et al., 2004). A point here, crucial to this paper's contentions, is the preeminence of the entrepreneur's role, given that it is he/she that must convince these existing controllers of resources to apply them to the newly proposed use, which according to Hellman (2007) is a daunting task. This begs the question: do investors place their bets on the entrepreneurial idea, or on the entrepreneur behind the idea?

Whilst certain scholars claim that the ‘idea’ – the business plan - is what matters most (Mason and Stark, 2004; Bamberger, 1994), since the business plan is the first and often substantial contact that the potential investor has with the entrepreneur (Shepherd and Douglas, 1999; Barrow et al., 2001: p6), there are others who argue otherwise: ‘what is a business plan without a businessman?’ (Driessen and Zwart, 2006). These scholars -amongst several others- whilst arguing that the greatest determinant of a business's success is the entrepreneur him/herself, further state, that although the business knowledge and craftsmanship of the entrepreneur are indeed important, what albeit merits even greater consideration, is the personality of the entrepreneur. Extending this further, are Nunes et al. (2014), who maintain that besides the criteria most valued by VC's being the entrepreneur's personality, is the quality of the management team. In like vein, Macmillan et al. (1985) vociferously claim that:

*‘…..above all it is the quality of the entrepreneur that ultimately determines the funding decision’.*

According to these scholars, five of the top ten criteria considered by VC's as being most important in entrepreneurs, concern the entrepreneur's experience or personality. They further state: ‘There is no question, that irrespective of the horse (product), horse race (market), or odds (financial criteria), it is the jockey (entrepreneur) who fundamentally determines whether the venture capitalist will place a bet at all’.

Given the aforesaid influence that the entrepreneur's personality and qualities wield on investors' investment related decisions, we argue that there is a strong case for entrepreneurs assessing their personalities in terms of brands, and for investing their time and efforts on personal brand building activities, so as to enhance their chances of attracting investments, based on their personal brands' increased credibility.

The entrepreneurship literature significantly addresses entrepreneurs' personalities and traits, with much emphasis on the characteristics of successful entrepreneurs: Need for Achievement (n Ach) (Mc Clelland, 1961; Perry et al., 1986; Begley and Boyd, 1987), Internal Locus of control (Ahmed, 1985; Hood and Young, 1993; Begley and Boyd, 1987; Gatewood et al., 1995) and Risk Taking Propensity (Dart, 1971; Meyer et al., 1961; Liles, 1974). Others, such as Littunen (2000) have examined the impact of entrepreneurship on the entrepreneur's personality, and yet others (see Mitchell et al., 2002; Coulton and Udell, 1976), the contributions of the entrepreneur's personality to new venture formation, and finally, Miller (2015) and DeNissi (2015), the personality attributes that eventuate in entrepreneurial failures.

A conspicuous, serious omission in the foregoing however, is the disregard for the critical role of the entrepreneur's personality in attracting investment, and the notion of entrepreneurs developing their personality brand competitiveness -through brand building strategies- for reasons discussed earlier. This is a gap that needs addressing.

Despite at least 95% of all businesses being SME's, branding has traditionally been considered a large companies' issue, lacks an SME perspective (e.g. Krake, 2005; Wong and Merrilees, 2005; Berthon et al., 2008), and has been rarely studied by SME's (Ahonen, 2008). Whilst branding -from an organizational perspective-has been defined as a programmatic approach to the selling of a product, service, organization, cause, or person, that is fashioned as a proactive response to the emerging desires of a target audience or market (Lair et al., 2005) the first use of the term ‘personal branding’ is attributed to Tom Peter's (1997) article in the magazine Fast Company.

The significance of entrepreneurs' personal branding is best understood in light of the argument of Lair et al. (2005), that personal branding goes beyond a simple and necessary strategy for individuals to negotiate a turbulent economic environment. Corroborating their stance are others such as Christensen and Cheney (2000, p. 246) according to whom: “*The market of today seems to be demanding well-crafted identities, identities that are able to stand out and break through the clutter. Because branding is so well suited to present images as identity, branding as a strategy has become increasingly important as a flexible response to a crowded communication world*”. In like vein, Arruda (2002, p.5) claims: *“Gone are the days where your value to your company or clients is from your offerings alone. Today, people want to buy brands–unique promises of value”*.

Ironically, despite the significant benefits that entrepreneurs derive from indulging in personal branding initiatives, this facet has been neglected within the mainstream entrepreneurship literature (Raftari and Amiri, 2014). Although the marketing literature carries a relatively greater share of this strand of literature, the bulk of how exactly to brand one's self for business world success, occurs more in non-academic books, magazines, web sites, training programs, and commentaries of personal coaches (Boyle, 2003; Ahonen, 2008). Also importantly, whereas the strands within the entrepreneurship literature on entrepreneurial identity and legitimacy also concern themselves with elements of the entrepreneur's personality in the context of new venture plausibility (Navis and Glynn, 2011; Middleton, 2013; Kibler and Kautonen, 2014), neither of these strands -with their multi-level foci-concentrate solely on the entrepreneur, nor on the notion of entrepreneurial branding in attracting funding, which happen to be the chief concern of this study.

Given the research paucity on the role and influence of the entrepreneur's personality brand on the investment decision criteria, this study seeks to fill this gap by quantitatively and qualitatively testing the validity of Entrepreneurial Brand Personality Equity model (of Balakrishnan and Michael, 2011) and also determining how the entrepreneur's different personality dimensions have an impact on the financing decisions of different types of investors, and at varying stages of the business.

## 3 Methodology

### 3.1 Design

**Fig. 1:**
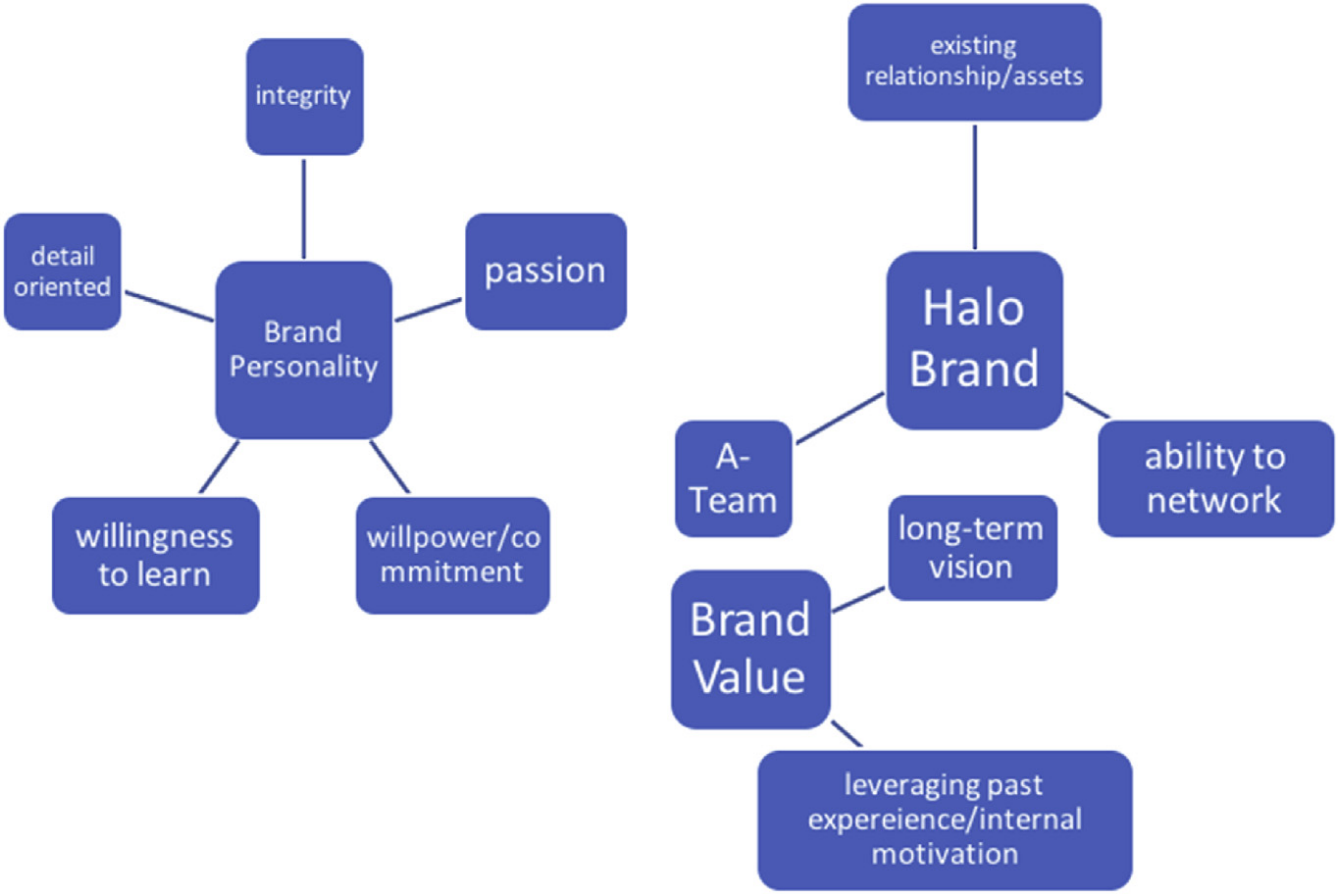
EBPE model. Source: Constructed by the authors.

As earlier discussed, we test the validity of the EBPE model, to confirm the importance of entrepreneurs' personal branding in attracting finance. Fig. 1 shows the EBPE model as constituting three key dimensions: Brand Personality (BP), Halo Brands (HB), and Brand Value (BV). Each dimension is associated with a set of variables. Whereas the BP dimension comprises variables including integrity, passion, confidence, detail oriented, commitment, willpower, overcoming fear of failure, and willingness to learn, the HB dimension comprises relationship assets, the ability to network, and the role and impact of teams, and the BV dimension -that is associated with the future potential of the entrepreneur- comprises the following: long term vision, ability to leverage past experiences and internal motivation, and the ability to be a critical judge.

### 3.2 Hypotheses

Consistent with our research objectives we test the following hypotheses.

H0 **EBPE variables are equally valued by different types of investors**

H1 **EBPE variables are not equally valued by different types of investors**

H00 **EBPE model's variables are equally valued at different stages of the business**

H2 **EBPE model's variables are not equally valued at different stages of the business**

### 3.3 Methods and data

Whilst the study uses a survey as a quantitative tool to collect data on the investors' perceptions of the EBPE model's dimensions, the qualitative part constitutes interviews, to further clarify and gain deeper insights into investors' views, that hold implications for the entrepreneur's ability to sustain the capability to attract their (investors') finance. Answers from both sources of data were integrated to verify the consistency of findings, that would lead to rich, robust, comprehensive and validated results of our study.

The survey was structured into three sections that respectively addressed the following aspects: Demographic^3^; Firmographic^4^; and the EBPE model's dimensions assessed on a 5-point Likert scale.

The investors' population comprised AI's, VC's, and PEI's. We identified our sample from three of the largest investor internet directories (*Angel List, Crunch Base and VC Gate).* We used stratified random sampling to identify 2750 investors, based on the highest number of investments made as listed in their profiles and with the aim to have a balanced number of AIs and VCs. Although the Angel List and Crunch Base populations are substantial, they are mostly limited to the regions with the most significant VC activity (Silicon Valley, Boston, Southwest of USA). The most challenging part was obtaining the investors' direct email addresses to compile the mailing list, which entailed researching each individual from Internet search engines. This reduced the final sample to 446 investors (further details are below).

For the interviews, we used opportunity sampling^5^ (personal contacts) for selecting interview participants. Ten semi-structured interviews were held with a mix of AI's and VC's operating in different parts of the world. The aim of this was to obtain their opinions from different angles, normally not feasible through a structured survey.

For the purpose of testing the reliability of the survey instrument, we sought assistance from a reputed, vastly experienced quantitative research consultant to complete an ‘expert driven pretest’ of the same (see Presser and Blair, 1994), in order to identify potential problems with questions, or response options within the survey. We adopted this approach owing to the logistical constraints involved with piloting the survey by directly contacting respondents^6^. Besides this, the pilot questionnaire was also sent for feedback on clarity of wording, survey structure and other possible omissions, to several academic-researcher colleagues as well as business persons familiar with the topic.

After trialing the survey, a brief study description was emailed to the target group [of 446], along with a consent form and confidentiality letter. 21 of the 446 investors declined our request to take the survey, and were hence removed from the sample. The survey was emailed to the remaining 425 investors, which elicited a response rate of 20.9%; that equaled 89 complete responses, 19 incomplete responses, 60 ‘opted out’ cases, and 257 cases wherein no responses were received. It is worth mentioning here, that the 89 complete responses received were slightly lower than the expected number, despite concerted efforts to improve upon the response rate. We however overcame this handicap by enhancing the depth and breadth of the topics discussed with interviewees, as part of the qualitative data gathering process, which is discussed next.

For interviews, the interviewees were assured of the confidentiality of interview data, and briefed in detail regarding the nature of the study, and the survey questions comprising the interview. Whilst a few interviewees were met at their offices, there were others who were interviewed at mutually convenient locations (such as coffee shops), and also over the phone. The aforesaid data was collected during the period 2011 and 2018.

## 4 Results

### 4.1 Descriptive analysis: demographics and firmographics

**Fig. 2:**
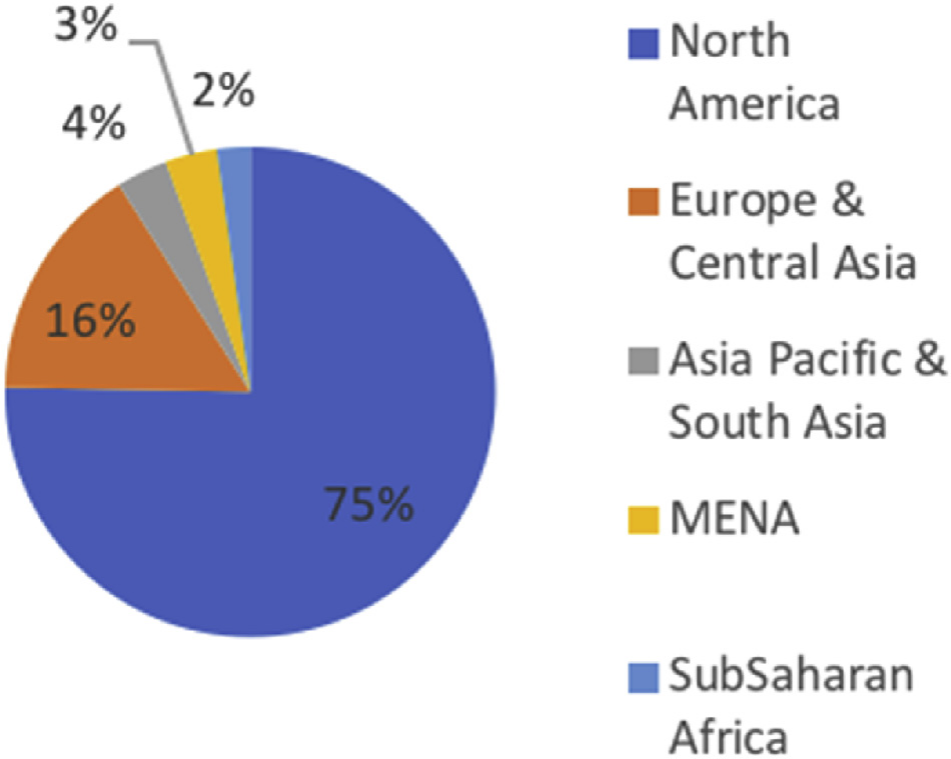
Location of business.

The majority of the sample's respondents (69.7%) were US investors^7^, followed by European investors (22.2%). As regards the location of businesses, North America attracted more than 75% of the capital investment of all respondent investors as shown in Fig. 2.

Three types of investors constituted our sample: 59.6% of VCs, followed by 31.5% AIs, and only 9% who are PEI's investors^8^.

**Fig. 3:**
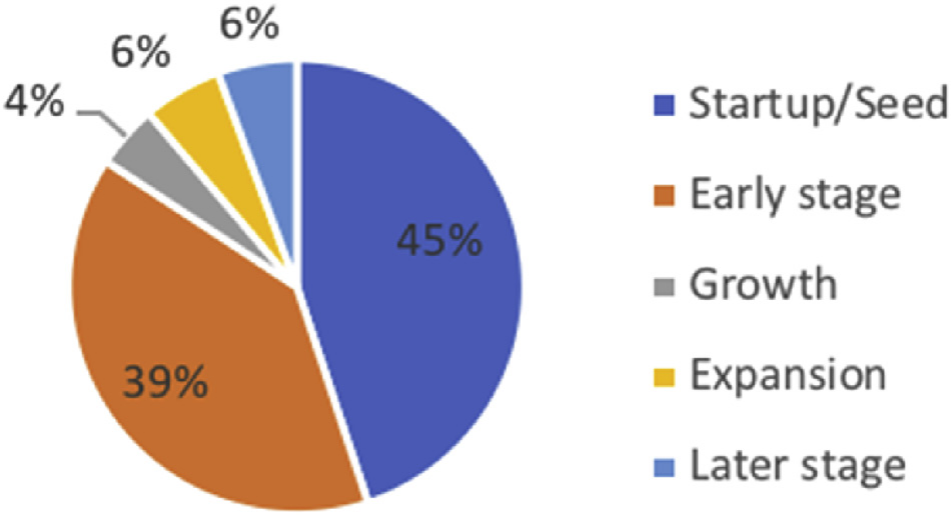
Stage of business at investment time.

**Fig. 4:**
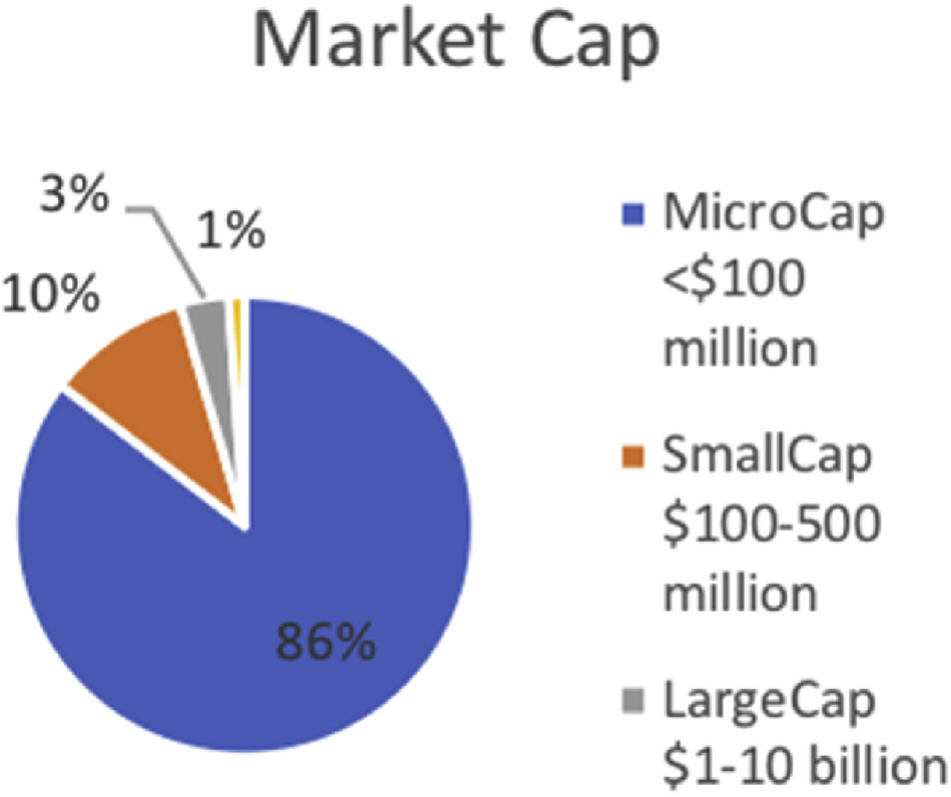
Size of business financed (Market Cap).

Fig. 3 depicts a high demand by investors to finance Startup/Seed and Early stage funding (85%). Likewise, 85% of the investors in our sample invested in micro-capital companies^9^ as presented in Fig. 4.

### 4.2 The importance of EBPE variables for investment decisions

All investors' responses of 3 and above on the Likert scale rating for EBPE variables, are considered as admitting their importance (Macmillan et al., 1985) and thus validating the EBPE model.

#### 4.2.1 Dimension 1: brand personality

**Fig. 5:**
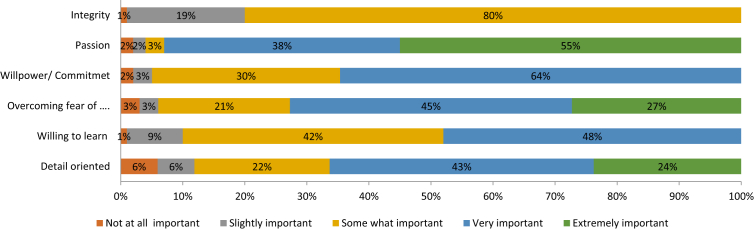
Importance of brand personality variables.

A majority of the respondents acknowledged the need for the BP variables, for investments to take place. Fig. 5 shows more than 90% of investors agreeing that Integrity, Passion, Willpower/Commitment and Willingness to learn are important to finance any business.

Whilst the other two variables were considered important too (and scored above 60%), the BP dimension was rated as being relatively more important.

The descriptives of the BP dimension in Table 1, indicate the central tendency of the frequency distribution as being skewed towards the ‘extremely important’ level for Integrity, Passion, Willpower/Commitment & Willingness to learn, and towards the ‘very important’ level for Confidence and Detail orientation. The relatively high standard deviation is explained by the variability in the observed data^10^. The overall responses are distributed very close to the mean values, resulting in a negatively skewed frequency distribution for all the variables, which indicate that the respondents assigned higher than the average scores^11^.

**Table 1:** Descriptives of BP variables.

		Integrity	Passion	Willpower/commitment		Over. fear of failure/confidence	Willing to learn	Detail oriented
N	Valid	89	89		89	89	89	89
	Missing	0	0		0	0	0	0
Mean		4.764	4.427		4.539	3.888	4.360	3.730
Std. error of mean		0.059	0.086		0.081	0.101	0.0787	0.113
Median		5	5		5	4	4	4
Mode		5	5		5	4	5	4
Std. deviation		0.56	0.810		0.770	0.959	0.742	1.063
Variance		0.319	0.657		0.592	0.919	0.551	1.131
Skewness		−3.876	−2.118		−2.507	−0.958	−1.377	−0.887
Range		4	4		4	4	4	4

*Note:* SPSS confidence interval for mean = 95%.

#### 4.2.2 Dimension 2: Halo brand

**Fig. 6:**
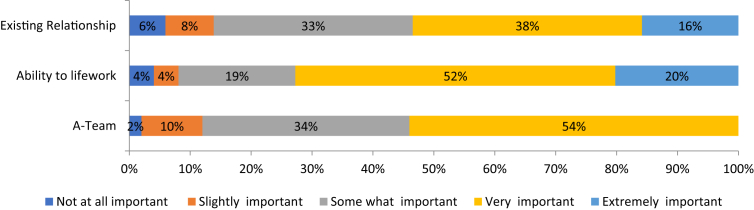
Importance of Halo brand variables.

The bar chart in Fig. 6 demonstrates the importance of the HB dimension to the investors. Here, 88% of the respondents indicated the *A-Team* as being a variable that must be present in order for an investment to take place, compared to the 72% who opted for *Ability to network*, and 54%, for *Existing relationships/assets*.

The descriptives of the HB dimension in Table 2, show that the mean, median and mode for the HB variables have central tendencies that are at the ‘extremely important’ level for the A-Team and at the ‘very important’ level, for Existing Relationships and Ability to Network.

**Table 2:** Descriptives of HB variables.

		Existing Rel./Assets	Ability to network	A-team
N	Valid	89	89	89
	Missing	0	0	0
Mean		3.506	3.787	4.371
Std. error of mean		0.110	0.103	0.090
Median		4	4	5
Mode		4	4	5
Std. deviation		1.035	0.971	0.845
Variance		1.071	0.943	0.713
Skewness		−0.582	−1.08	−1.724
Range		4	4	4

*Note:* SPSS confidence interval for mean = 95%.

Similarly, the relatively high standard deviation could be explained by the variations within the observed data. The distribution of the overall responses very close to the mean values, has resulted in a negatively skewed frequency distribution for all variables.

#### 4.2.3 Dimension 3: brand value

**Fig. 7:**
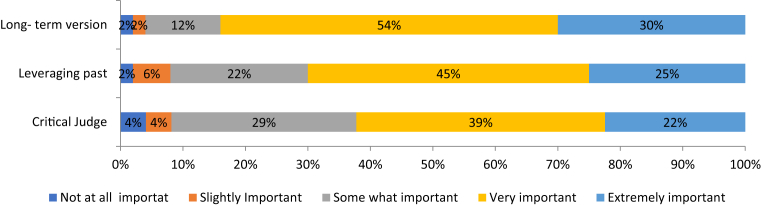
Importance of brand value variables.

The bar chart in Fig. 7 indicates the importance of the BV dimension for the investment decisions. Whereas 84% of respondent investors rated Long-term vision as a variable that must be present in order for an investment to take place, 70% and 61% of investors, reported the importance of Leveraging past experiences/Internal motivation, and Critical Judge respectively for any financing decision.

Data from Table 3 indicates that the mean, median and mode for BV variables too, have central tendencies that are at the ‘extremely important’ level for Long-term Vision, and at the ‘very important’ level for Leveraging past experiences/Internal Motivation and Critical Judge. Whilst the standard deviation is relatively high due to the variability in the observed data, the overall responses were distributed very close to the mean values, resulting in a negatively skewed frequency distribution for all the variables. The low standard error of mean relative to the mean for all EBPE variables, confirms that our sample is a good representative of the population.

**Table 3:** Descriptives of BV variables.

		Long-term vision	Leveraging past exp./Internal motiv.	Critical judge
N	Valid	89	89	89
	Missing	0	0	0
Mean		4.101	3.843	3.708
Std. error of mean		0.083	0.100	0.107
Median		4	4	4
Mode		4	4	4
Std. deviation		0.784	0.940	1.014
Variance		0.615	0.884	1.027
Skewness		−1.049	−0.768	−0.721
Range		4	4	4

*Note:* SPSS confidence interval for mean = 95%.

The aforesaid results verify that all variables of the EBPE model's dimensions are extremely important for the investment decision. This deduction is a confirmation, that the model's dimensions must be present for a positive investment decision, and could therefore be treated as a validiation of the EBPE model. This corroborates our views about the criticality of the entrepreneur's role in attracting finance.

### 4.3 Differences in the importance of EBPE's variables for different investors

Kruskal-Wallis test was the most appropriate test^12^ to determine if there are differences in the importance assigned to the EBPE's variables between the three groups of investors.

A Kruskal-Wallis test was run to determine if there were differences in the importance assigned to the EBPE's variables between the three groups of investors. This test was deemed the most appropriate, since it met all the required assumptions: there was one nominal variable with three independent groups (the categories of investors) and one dependent measurable variable (the Likert scale rating), each participant belongs to one category only, and data was not normally but similarly distributed (as seen in histograms of Appendix N, P and R; see Laerd, 2014). Mann-Whitney test was also considered (however it only applies to a maximum of two groups within the independent variable), and also the ANOVA test (that assumes normal distribution). Therefore both these tests were deemed unsuitable for this analysis.

Whilst this test normally computes ***median values and mean ranks***, the median test could not be performed in this analysis for all variables (due to insufficient valid cases) and therefore, the results have not been interpreted. The results of this test for the mean ranks in Table 4, reveal no statistically significant differences in most of the EBPE variables between the different investors groups. A-team was the only variable that recorded a statistically significant difference across the groups of investors, given results of χ^2^(2) and p > 0.05.

**Table 4:** Hypothesis H0 test summary.

	Null hypothesis	Test	Sig.	Decision
1	The distribution of integrity is the same across categories of the type of investor	Independent samples Kruskal-Walls test	.923	Retain the null hypothesis
2	The distribution of passion is the same across categories of the type of investor	Independent-samples Kruskal-Walls test	.764	Retain the null hypothesis
3	The distribution of willpower commitment is the same across categories of the type of investor	Independent-samples Kruskal-Walls test	.703	Retain the null hypothesis
4	The distribution of overcoming fear of failure/confidence is the same across categories of the type of investor	Independent-samples Kruskal-Walls test	.870	Retain the null hypothesis
5	The distribution of willing to learning is the same across categories of the type of investor	Independent-samples Kruskal-Walls test	.639	Retain the null hypothesis
6	The distribution of the detail oriented is the same across categories of the type of investor	Independent-samples Kruskal-Walls test	.754	Retain the null hypothesis
7	The distribution of existing relationships/assets is the same across categories of the type of investor	Independent-samples Kruskal-Walls test	.135	Retain the null hypothesis
8	The distribution of the ability to network is the same across categories of the type of investor	Independent-samples Kruskal-Walls test	.904	Retain the null hypothesis
9	The distribution of A-Team is the same across categories of the type of investor	Independent-samples Kruskal-Walls test	.040	Reject the null hypothesis
10	The distribution of Long Term Version is the same across categories of the type of investor	Independent-samples Kruskal-Walls test	.075	Retain the null hypothesis
11	The distribution of Leveraging Past Experiences/Internal Motivation is the same across categories of the type of investor	Independent-samples Kruskal-Walls test	.371	Retain the null hypothesis
12	The distribution of Critical Judge is the same across categories of the type of investor	Independent-samples Kruskal-Walls test	.371	Retain the null hypothesis

A symptotic significance are displayed. The significance level is 0.5.

Resultantly, ***the null hypothesis was “not rejected” for all EBPE variables except for the*** A-team, which is a variable in HB dimension. This result indicates that the association between the type of investors and their opinion of EBPE variables is likely to be explained by chance alone. However, the A-team was the only variable that recorded the null hypothesis rejection.

**Fig. 8:**
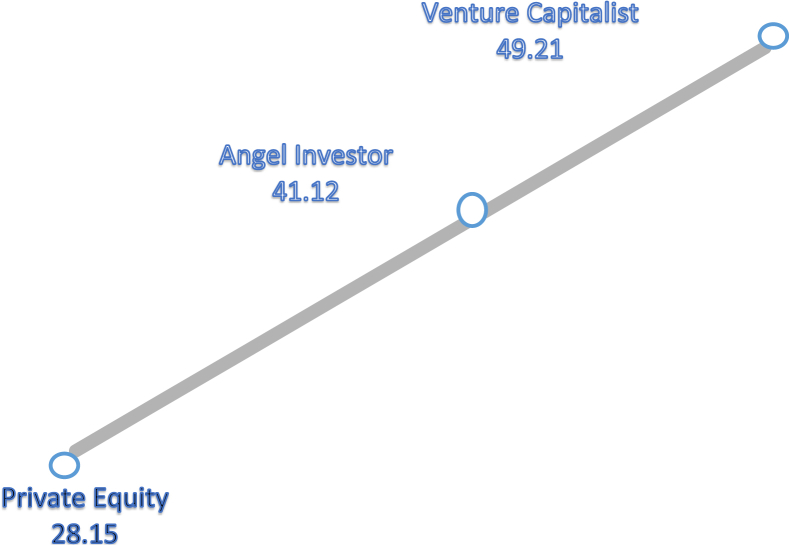
Pairwise comparisons of types of investors for A-team variable. *Each node shows the sample average rank of types of investors.

Further, since the Kruskal-Wallis test only indicates if the difference exists between groups, but does not specify which ones, we undertook a pairwise comparison among the three groups for the A-Team variable. The result of this test in Fig. 8 and Table 5 show a significant difference between VC's and PEI's in terms of their importance accorded to the A-Team variable (p-value = .017). The lowest node of PEI's indicates the low importance of the A-team for such investors, which is most likely due to the nature of their investment process.

**Table 5:** Pairwise comparisons of Types of Investors for A-Team variable.

Sample 1–sample 2	Test statistic	Std. error	Std. test statistic	Sig.	Adj.Sig.
Private equity-angel investor	13.571	9.286	1.461	.144	.432
Private equity-venture capitalist	20.958	8.786	2.385	.017	.051
Angel investor- venture capitalist	7.386	5.412	1.365	.172	.517

Each row tests the null hypothesis that sample 1 and sample 2 distributions are the same.

Asymptotic significances (2-sides tests) are displayed. The significance level is .05.

### 4.4 Differences in the importance of EBPE's variables for stages of business

The Kruskal-Wallis test was used to determine if there were differences in the importance assigned to the EBPE variables at different stages of the business at the time of investment^13^. The mean ranks were found to differ among various stages of business development, with higher values for startup/seed, early stage and expansion of the business, and lower for later stage, for all EBPE variables. The results in Table 6 do not show any statistically significant differences for most of the EBPE variables between the different business stages. Only two variables; A-team and Leveraging experience/Internal motivation, recorded a statistically significant association with the stage of business, given results of χ^2^(2) and p > 0.05.

**Table 6:** Hypothesis H00 test summary.

	Null hypothesis	Test	Sig.	Decision
1	The distribution of integrity is the same across categories of the type of investor	Independent samples Kruskal-Walls test	.848	Retain the null hypothesis
2	The distribution of passion is the same across categories of the type of investor	Independent-samples Kruskal-Walls test	.765	Retain the null hypothesis
3	The distribution of willpower commitment is the same across categories of the type of investor	Independent-samples Kruskal-Walls test	.194	Retain the null hypothesis
4	The distribution of overcoming fear of failure/confidence is the same across categories of the type of investor	Independent-samples Kruskal-Walls test	.999	Retain the null hypothesis
5	The distribution of willing to learning is the same across categories of the type of investor	Independent-samples Kruskal-Walls test	.320	Retain the null hypothesis
6	The distribution of the detail oriented is the same across categories of the type of investor	Independent-samples Kruskal-Walls test	.550	Retain the null hypothesis
7	The distribution of existing relationships/assets is the same across categories of the type of investor	Independent-samples Kruskal-Walls test	.073	Retain the null hypothesis
8	The distribution of the ability to network is the same across categories of the type of investor	Independent-samples Kruskal-Walls test	.111	Retain the null hypothesis
9	The distribution of A-Team is the same across categories of the type of investor	Independent-samples Kruskal-Walls test	.332	Retain the null hypothesis
10	The distribution of Long Term Version is the same across categories of the type of investor	Independent-samples Kruskal-Walls test	.036	Reject the null hypothesis
11	The distribution of Leveraging Past Experiences/Internal Motivation is the same across categories of the type of investor	Independent-samples Kruskal-Walls test	.185	Retain the null hypothesis
12	The distribution of Critical Judge is the same across categories of the type of investor	Independent-samples Kruskal-Walls test	.003	Reject the null hypothesis

A symptotic significance are displayed. The significance level is 0.5.

Accordingly, ***the null hypothesis was “not rejected” for all EBPE variables except for the*** A-team and Leveraging experience/Internal motivation, which are variables in HB and BV dimensions.

This result indicates that the association between the ***stages of business development and the importance of these EBPE variables*** is likely to be explained by chance alone. However, the A-team and Leveraging experience/Internal motivation were the only variables that recorded a rejection of the null hypothesis.

**Fig. 9:**
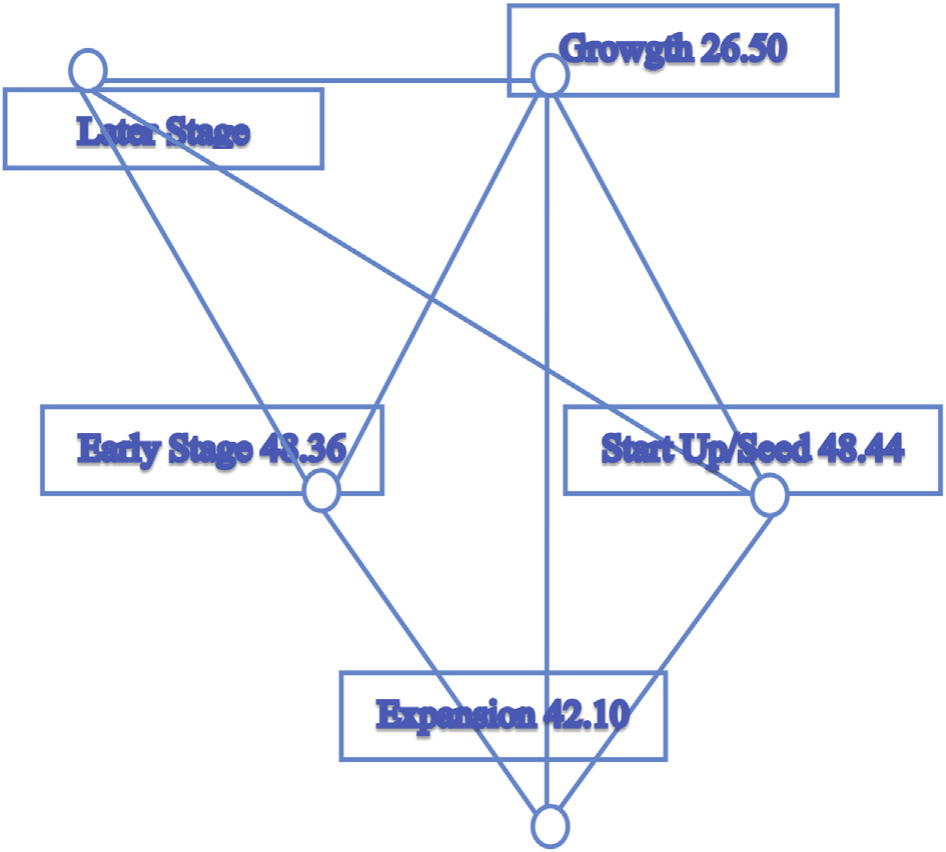
Pairwise comparisons of stages of business at investment time for A-Team. *Each node shows the sample average rank of stages of business at investment time.

We applied a pairwise comparison among the the stages of business development for the A-Team variable. The results in Fig. 9 and Table 7 indicate a statistically significant difference^14^ between Startup/Seed and Early Stage, and Later Stage in terms of importance assigned to the A-Team variable.

**Table 7:** Pairwise comparisons of Stages of business at investment time for A-team.

Sample 1–Sample 2	Test statistic	Std. error	Std. test statistic	Sig.	Adj.Sig.
Later stage-growth	16.700	15.539	1.075	.283	1.000
Later stage-expansion	32.300	14.650	2.205	.027	.275
Later stage-start up/seed	38.638	10.988	3.516	.000	.004
Later stage-early stage	38.829	11.075	3.506	.000	.005
Growth-Expansion	−15.600	15.539	−1.004	.315	1.000
Growth-start up/seed	21.938	12.148	1.806	.071	.709
Growth-early stage	22.129	12.226	1.810	.070	.703
Expansion-start up/seed	6.338	10.988	.577	.564	1.000
Expansion-early stage	6.529	11.075	.590	.556	1.000
Start up/seed-early stage	−.191	5.362	−.036	.972	1.000

Each row tests the null hypothesis that sample 1 and sample 2 distributions are the same.

Asymptotic significances (2-sides tests) are displayed. The significance level is .05.

**Fig. 10:**
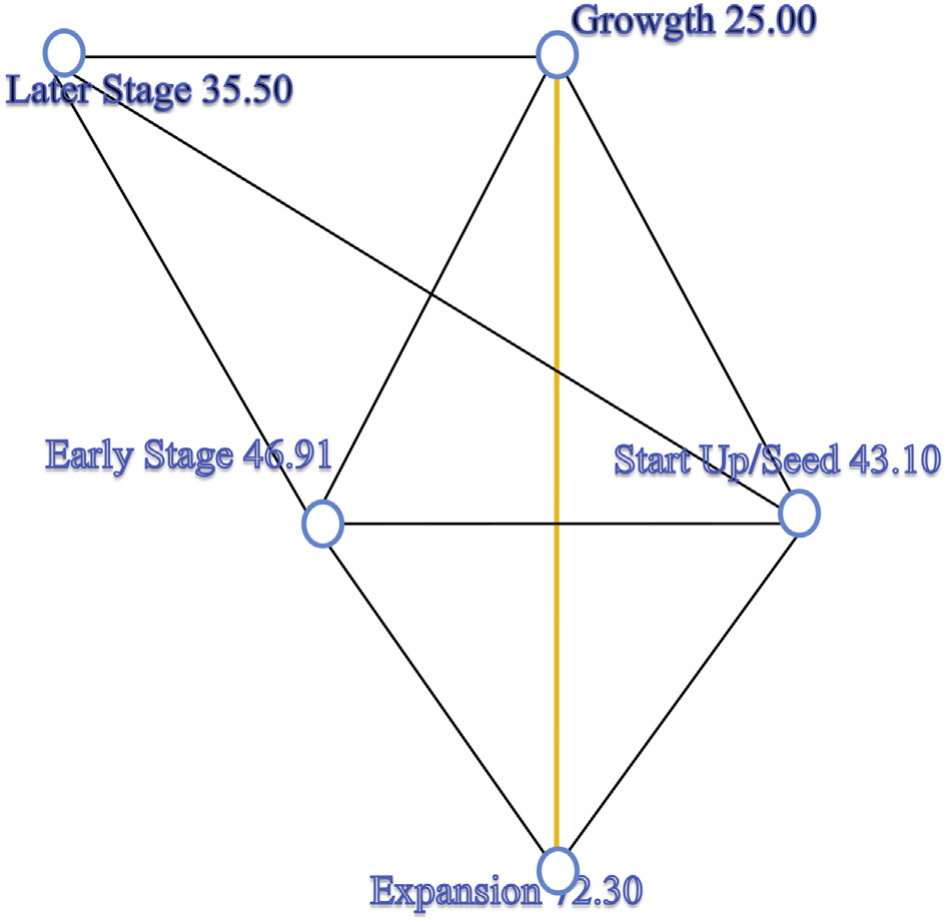
Pairwise comparisons of stages of business at investment time, for leveraging past experience/internal motivation variables. *Each node shows the sample average rank of stages of business at investment time.

The results of the pairwise comparison among the stages of business development for the Leveraging past experience and Internal motivation variable, in Fig. 10 and Table 8, reveal significant differences between the Growth and Expansion stages in terms of the importance assigned to Leveraging past experience and Internal motivation (p-value = 0.04).

**Table 8:** Pairwise comparisons of stages of business at investment time, for leveraging past experience/internal motivation variables.

Sample 1–sample 2	Test statistic	Std. error	Std. test statistic	Sig.	Adj.Sig.
Later stage-growth	−10.500	16.283	−.645	.519	1.000
Growth-start up/seed	18.1000	12–729	1.422	.155	1.000
Growth-early stage	21.914	12.811	1.711	.087	.872
Growth-expansion	−47.300	16.283	−2.905	.004	.037
Later stage-start up/seed	7.600	11.514	.660	.509	1.000
Later stage-early stage	11.414	11.605	.984	.325	1.000
Later stage-expansion	36.800	15.352	2.397	.017	.165
Start up/seed-early stage	−3.814	5.618	−.679	.497	1.000
Start up/seed-expansion	−29.200	11.514	−2.536	.011	.112
Early stage-expansion	−25.386	11.605	−2.188	.029	.287

Each row tests the null hypothesis that sample 1 and sample 2 distributions are the same.

Asymptotic significances (2-sides tests) are displayed. The significance level is .05.

Further, we held several interviews and performed a content analysis of the results using NVivo qualitative data analysis software, to obtain deeper insights into investors' thinking, as regards the model's variables that they felt were important for entrepreneurs to possess, in order to ensure the continued financing of their ventures by the investors.

The interviewees confirmed that the personal factors outweighed all other criteria, such as size of market or business idea, and felt that the absence of these could lead to business failure. They opined that a company at its inception is just a blank piece of paper, and they could only invest in the people (entrepreneurs) who became “the single biggest risk factor as well as success factor of the business”. One of the respondents commented thus:

*“What I have learned after reviewing and also meeting with several startups and investments, is there is no formula for investment at the seed stage because there are no numbers and tractions for us to look at, but the A-team is everything If you can measure what they are speaking about and express it in numbers, then they are up to something”*

Investors consider entrepreneurs as being represented by a set of values that make up their personal brand, which ultimately defines the future corporate brand. We quote one of the interviewees:

*“The persons who found the company, by definition, bring their value systems, their attitudes, their mentalities to the company, and that company is then shaped by their personal brand, and it becomes a kind of a product of their value system”*

An interesting corollary to this aspect that also emerged, was the need for striking a balance between personal and corporate brands, to avoid falling prey to the ‘Founder's Syndrome’, which is about organizations' growth being stifled by dependence on their founders, who in part, owing to their emotional connection to their ventures, cannot identify or address weaknesses within the same (Shortall, 2007). These views are borne out by Schein's seminal work (1983) on the role and impact of founders on their firms'cultures. A further elaboration and conclusions of the foregoing results are presented below.

## 5 Discussion and conclusion

As access to finance has been a ‘perennial’ problem for entrepreneurs, they often approach the relatively few AI's, VC's and PEI's, to fund their ventures. What flows from this is whether the VC's, AI's and PEI's fund entrepreneurs' ideas, or those behind the ideas. In this article we have reasoned that investors accord greater importance to entrepreneurs than to their ideas, and have drawn on arguments of several scholars to argue for the influence of the entrepreneur's personal brand on the investors-during their investment related decision making processes-an area under researched in the entrepreneurship literature.

According to Cohen and Kador (2013) *“There is a big difference between a good business and a good investment. The difference is always the entrepreneur”.* This study has extended this line of thinking further, in arguing that what makes an even greater difference, is the entrepreneur's brand.

Since its conception in 2011, the EBPE model's efficacy from an investors' perspective- in relation to actual investments undertaken based on entrepreneurs' personalities- was not ascertained. We hope that our validation of the model facilitates the adoption (by entrepreneurs) of personal brand building strategies most suited to initially attracting investors' funding, and to subsequently ensure the sustenance of such financing arrangements.

Overall, our study's results support the importance of the EBPE model (in its entirety) to investors' decision making, as regards their amenability to financing entrepreneurial ventures. The overall descriptive results^15^ of the EBPE model, revealed that all variables of the BP, HP and BV dimensions were necessary for an investment to take place, thus supporting the validity of the model. Six variables emerged as the most critical ones: Integrity, Passion, Willpower/Commitment, Willingness to learn, A-Team and Long-term Vision, and no other factors could specifically compensate for their absence.

Further, the results of the non-parametric tests suggests that the A-Team excepted, all the EBPE model's variables have been perceived similarly by all types of investors (VCs, AIs and PEIs). This indicates an insignificant correlation between the types of investors and their opinions about most of the model's variables. The A-Team however was an exception, hence perceived differently depending on the type of the investor. A further investigation revealed a significant difference between VC's, AI's and PEI's, in terms of the importance they assigned to the A-team variable. Relative to the importance attached to this factor by VC's and AI's, the PEI's gave the least consideration to the A-Team. However, this latter occurrence is since PEI's, besides being better suited to reviving weak companies than financing entrepreneurial, innovative, new ventures, are known for taking a controlling position in businesses (Dutia, 2012), and beyond financing, provide strategic expertise and support to the company's management, thereby nullifying the need for strong A-Teams (Capman, n.d.).

Furthermore, with the exception of two variables (the A-Team, and Leveraging experience/Internal motivation), all investors accorded the same level of importance to the model's variables, at all stages of the business. The A-Team was valued significantly higher in the Startup/Seed and Early stage, than in the later stage of business, whereas Leveraging past experience/Internal motivation was found to be valued significantly higher in the Expansion stage, than in the Growth Stage of business. This logically would mean, that in cases wherein an investor is contemplating investing in either of two businesses-one at the Later and the other the Start-up stage respectively-the investment would be made on the business with the better A-Team (or, put differently, the decision would be influenced by the quality of the A-Team of the ventures).

Similarly, in cases wherein an investor is contemplating investing in either of two businesses, one at the Later, and the other the Early stage respectively, the investment would -in this case too-be made on the business with the better A-Team (or, the decision would again be influenced by the quality of the A-Team of the ventures).

Finally, in cases wherein investors contemplate investing in either of two businesses-one at the Growth and the other at the Expansion stage respectively-the investment would be made on the business with the better A-Team (or, the decision would be influenced by the quality of the A-Team of the ventures).

The A-team clearly emerged as a critical variable of the EBPE model, as there often is a team -not just one person- behind the idea, and also since team members may have different values, attitudes, beliefs, different cultures, backgrounds, opinions and goals, with such diversity aiding to increase the venture's adaptive capability, by leveraging off the strengths of each member. This resonates well with the research findings on the broad topic of why diverse teams are smarter (see Rock and Grant, 2016).

Our quantitative results revealed that the A-team factor is significantly associated with the stage of business when deciding to invest, and it is valued significantly higher in the Startup/Seed and Early stage, than in the later stage of business. Leveraging past experience/Internal motivation is a factor that was found to be more important in the Expansion stage than in the Growth Stage of a business. The findings also showed that different types of investors do not assess all the dimensions differently, except for the A-Team, which was not an important factor for PEI's investment decisions.

Therefore, the above results indicate that if an investor chooses to finance several entrepreneurial ventures that are at different stages of business, and wherein the entrepreneurs/owners of each venture possess varying levels of experience/internal motivation, the investor's choices would be affected by the levels of experience/motivation when he/she chooses between projects at the growth, or at the expansion stage. The investor would choose a project at the expansion stage if the entrepreneurs possessed a sufficient level of experience/motivation, so as to maintain the expansion, and ensure the sustainability of the business. However, experience/motivation do not play a significat role in investment decisions relating to other stages of business.

Much of the data gathered and subsequent analysis performed for this study (through quantitative means) has been geared towards unravelling what investors sought in entrepreneurs' personalities, to make an investment. The qualitative component however, has been skewed more towards attempting to understand what investors would further seek in entrepreneurs, in order to continue investing in their ventures beyond the initial stages. The key themes that emerged from the interviews in this regard are as follows:

a) Regarding founders' (entrepreneurs') values:
  • Most companies are driven by a ‘founding’ kind of value system, and the value system of the founder defines the corporate brand.
  • The values of founders must be authentic to succeed.
  • If the integrity and value system are not as expected, then the business would fail.
b) Regarding the primacy of the A-Team:
  • With ‘no numbers’ for investors to rely upon at the seed stage and early stage, the A-Team of the new venture significantly influences investors' decisions.
  • The role of the A-Team is significantly higher in the Startup/Seed and Early stage, than in the later stage of business.
  • AI's and VC's in particular, seemed to place a higher premium on the A-Teams, relative to PEI's.
c) Regarding the role and importance of personal brands:
  • Building a personal brand and relationships are especially important in early stage companies, when the entreprenuer plays a predominant role. However, during the later stages, this matters more to employees, and public forums, and also the industry. These contentions concur with previous studies of scholars such as Rode and Vallaster (2005); Petty and Gruber (2011); Bresciani and Eppler (2010); and Eggers et al. (2013).
  • As the company grows, the focus must shift more from the personal towards the corporate brand, in the interest of the company's future.
  • In the absence of this shift, there is a danger that the entreprenuerial venture may become subordinated to the personal brand (or a prisoner of the entrepreneur's personality) that would threaten the sustainability of the business. This argument is cemented by the work of various scholars, (e.g., Shortall, 2007; Schein, 1983; Block and Rosenberg, 2002).

Overall-and within the context of investors' financing related decision making processes- our findings have revealed the importance of entrepreneurs' personal brands, their personal traits that are transferred to the business environments and future corporate brands, and their predominant role particularly in the early stages of business. These conclusions are consistent with the findings in the existing literature concerning the entrepreneur's or founder's common traits most valued by investors (see Nunes et al., 2014; Duening and Metzger, 2014; Caliendo et al., 2014; Silva, 2004; Miloud et al., 2012; Rose, 2014).

## 6 Related work

‘Entrepreneurship research has become so homogenized that it targets a very small audience of researchers, despite generating a dazzling variety of findings that are, unfortunately, barely connected to reality’ (Schultz, 2010). At odds with this are the results of this research, that hold significant implications for various stakeholders, and provide nascent entrepreneurs and newly established SMEs with critical insights on how best to utilize -and develop-their personal brands, to better influence their capital-seeking endeavors. Simultaneously, they aid investors in taking more informed investment decisions, by providing them with a framework to better assess entrepreneurs behind their respective ideas; and practitioners (such as brand consultants, marketers, business owners, and investors), to better orient and influence their decisions towards personal and corporate branding, so as to obtain superior outcomes. Whilst this research confirms earlier studies' findings regarding the broad acceptance of the importance of an entrepreneur's A-team, a specific message it offers to practitioners is regarding the significant difference between VC's, AI's and PEI's, in terms of the importance they assigned to the A-team, across various stages of a business.

The foregoing aspects -despite their significance for entrepreneurs and investors-are seemingly under researched topics within the entrepreneurship literature. An area for further research therefore, could be an exploration of whether different industry sectors demand other personal characteristics of entrepreneurs, in which case, a suitable modification of the EBPE model would be necessitated. Similarly, in the context of varying geographic locations, more studies would be worth undertaking, on cultural differences among investors and entrepreneurs, and their impact on investment decisions.

## Declarations

### Author contribution statement

Suzanna ElMassah, Ian Michael, Reynold James, Ionica Ghimpu: Conceived and designed the experiments; Performed the experiments; Analyzed and interpreted the data; Contributed reagents, materials, analysis tools or data; Wrote the paper.

### Funding statement

This research did not receive any specific grant from funding agencies in the public, commercial, or not-for-profit sectors.

### Competing interest statement

The authors declare no conflict of interest.

### Additional information

No additional information is available for this paper.

## Footnotes

2 The details of the the EBPE model are shown later in the conceptual framework.

3 Questions for the investors (regarding nationality, age, gender and investor type).

4 Questions regarding investment's location and sector, stage of business at the time of investment, investment volume by the investor, and the current state of the business).

5 This was owing to difficulty faced in gaining direct access to investors.

6 Mainly U.S.A. based.

7 USA is the biggest and the most active venture capital market and private investments in the world (Vanham, 2015).

8 Private equity is defined as a separate type of funding which involves investing very large amounts of money in mature businesses, which are restructuring through management or institutional buyouts (IBO). This study regards private equity as buyout firms.

9 Market-cap ranges used in the questionnaire are for business market-cap in general across all markets types. no further analysis of this data will be undertaken.

10 E.g., for Integrity, the 1% rating as ‘not at all important’ has clearly shifted the mean value & increased the SD.

11 However a negative skewness of <-3 can be a concern in terms of reliability of further testing.

12 The data is not normally distributed, rather, they are similarly distributed. Mann-Whitney test was not used, since it only applies to a maximum of two groups within the independent variable, and ANOVA test was not suitable, since it assumes normal distribution.

13 As mentioned previously, the median test could not be performed in this analysis for all variables due to insufficient valid cases, the results have hence not been interpreted.

14 P-value = 0.004 and 0.005.

15 Despite the asymmetric frequency distribution.
